# The Semaphorin 3A inhibitor SM-345431 preserves corneal nerve and epithelial integrity in a murine dry eye model

**DOI:** 10.1038/s41598-017-15682-1

**Published:** 2017-11-14

**Authors:** Risa Yamazaki, Katsuya Yamazoe, Satoru Yoshida, Shin Hatou, Emi Inagaki, Hideyuki Okano, Kazuo Tsubota, Shigeto Shimmura

**Affiliations:** 1 Department of Ophthalmology, Keio university school of medicine, Tokyo, Japan; 2 Department of Physiology, Keio university school of medicine, Tokyo, Japan; 3 Centre for Rare Disease Research, National Institutes of Biomedical Innovation, Health and Nutrition, Osaka, Japan

## Abstract

Dry eye disease (DED) is a common disorder causing discomfort and ocular fatigue. Corneal nerves are compromised in DED, which may further cause loss of corneal sensation and decreased tear secretion. Semaphorin 3A (Sema3A) is expressed by the corneal epithelium under stress, and is known as an inhibitor of axonal regeneration. Using a murine dry eye model, we found that topical SM-345431, a selective Sema3A inhibitor, preserved corneal sensitivity (2.3 ± 0.3 mm versus 1.4 ± 0.1 mm in vehicle control, p = 0.004) and tear volume (1.1 ± 0.1 mm versus 0.3 ± 0.1 mm in vehicle control, p < 0.001). Fluorescein staining area of the cornea due to damage to barrier function was also reduced (4.1 ± 0.9% in SM-345431 group versus 12.9 ± 2.2% in vehicle control, p < 0.001). The incidence of corneal epithelial erosions was significantly suppressed by SM-345431 (none in SM-345431 group versus six (21%) in vehicle control, p = 0.01). Furthermore, sub-epithelial corneal nerve density and intraepithelial expression of transient receptor potential vanilloid receptor 1 (TRPV1) were significantly preserved with SM-345431. Our results suggest that inhibition of Sema3A may be an effective therapy for DED.

## Introduction

The cornea protects the inner structures of the eye from external stimuli with a highly sophisticated defence system. Dense nerve fibres in the cornea provide quick blink reflexes and lacrimation that play important roles in protecting the eyes. The cornea is innervated by the ophthalmic nerve, the sensory division of the trigeminal nerve. Nerve endings suffer damage by a variety of disease such as trauma, surgical intervention, microbial infection, diabetic mellitus, and dry eye disease. Depending on the extent of neuronal damage, corneal sensitivity attenuates, and the tear secretion decreases^1,2^.

Dry eye diseases (DED) is a common, multifactorial pathology with an estimated prevalence of 14–30% of the population^3–6^. Recently, damage to the corneal afferent neurons in DED patients has been reported to be associated with the disease^7–9^. Tear film instability and inflammation cause physiological stress to the epithelium that leads to the release of danger-associated molecular pattern molecules (alarmins) and regeneration-associated genes (RAGs) that can affect axon guiding and nerve regeneration^10^. Semaphorin 3A (Sema3A) is a member of the semaphorin family of RAGs that are chemorepulsive guidance molecules that repel axons and collapse growth cones^11,12^, and as a result, inhibit axonal regeneration^13,14^. In the cornea, Sema3A acts as a repellent for nerves during the embryonic stage. Sema3A knockout mice have abnormal nerve innervation into the central cornea and lens, which are not observed in wild type mice^15^.

Sema3A is also expressed in the adult corneal epithelium, and excessive expression of Sema3A in corneal epithelial cells of rabbits causes repulsion or inhibition of the regeneration of A-delta and C nerve fibres^16^. Selective inhibitors of Sema3A were shown to enhance axon regeneration and motor function recovery in a spinal cord injury model^17,18^. Our group previously reported that the Sema3A inhibitor SM-345431 (vinaxanthone) promotes regeneration of corneal nerves and recovers corneal sensation in a murine keratoplasty model^19^.

In this study, we hypothesized that inhibiting Sema3A may protect afferent nerves from damage due dry eye. We therefore examined the effect of SM-345431 on corneal epithelial sensation, corneal epithelial barrier function, tear volume and corneal epithelial nerve density in a murine dry eye model.

## Results

### The Sema3A inhibitor SM-345431 preserves corneal epithelial integrity

Murine DED models were made by removing the right extra-orbital lacrimal glands. Mice were then divided to two groups, DED mice treated with phosphate-buffered saline (PBS) (Vehicle group), and DED mice treated with SM-345431 (0.5 mg/ml) (SM-345431 group). PBS (vehicle) or SM-345431 eye-drops were applied 3 times a day for 4 weeks immediately after lacrimal grand extraction.

To exclude the potential iatrogenic effects of the SM-345431, normal mice without lacrimal gland extraction (non-DED) were divided to three groups; untreated (no eye drops), vehicle, and SM-345431. Vehicle and SM-345431 eye-drops were applied 3 times a day for 4 weeks, while untreated mice did not receive eye drops.

**Figure 1:**
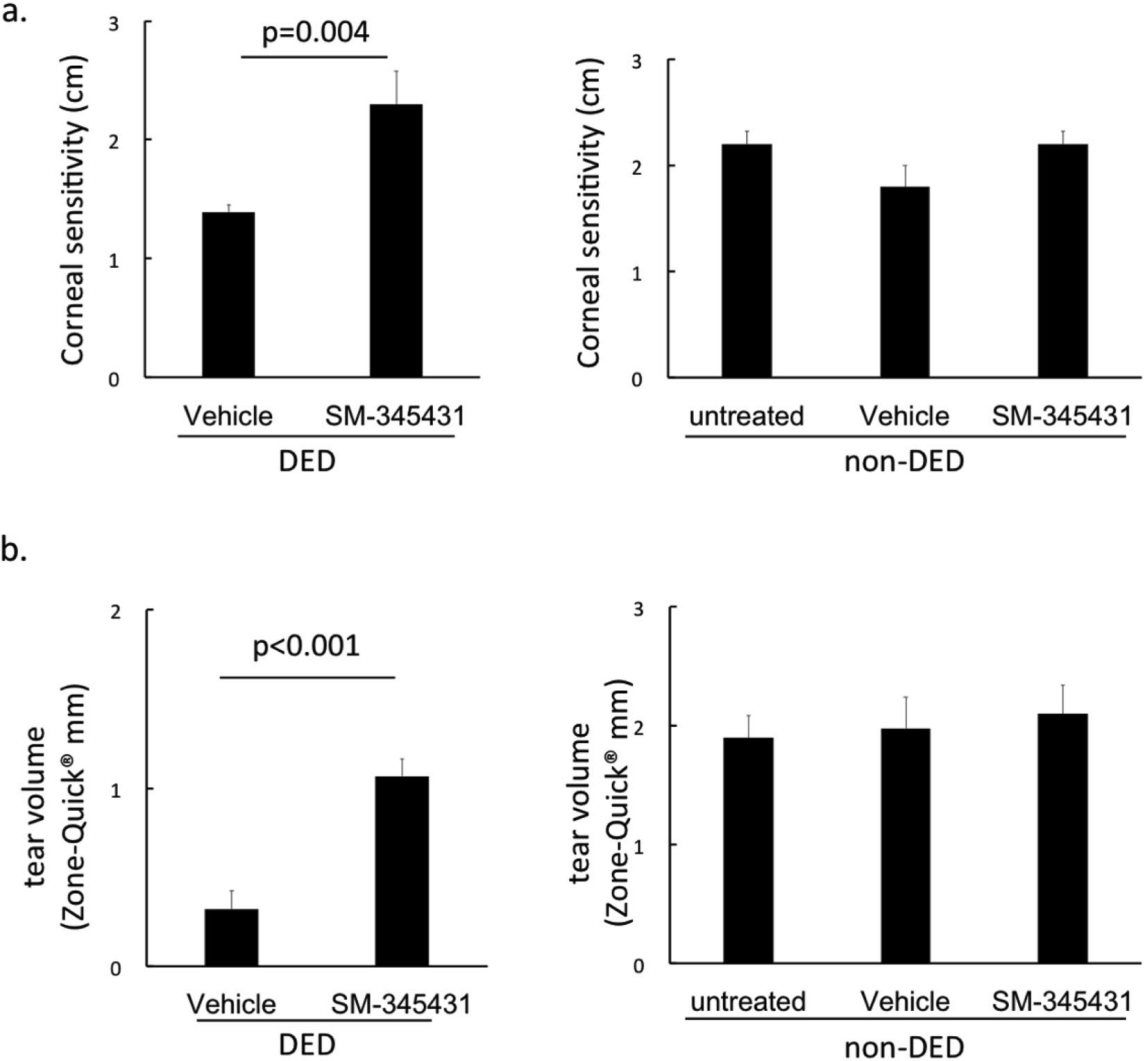
Corneal sensitivity and tear volume. (**a**) Corneal sensitivity after 4-week application of eye drops. DED group (left), and non-DED group (right). (**b**) Tear volume after 4 weeks of treatment. DED group (left), and non-DED group (right). DED groups were analysed by Student’s t-test, n = 15 mice/group. Intact groups were analysed by one way ANOVA and Bonferroni post hoc analysis, n = 5 mice/group. Graphs demonstrate as mean ± SD.

We measured corneal sensitivity, tear volume and corneal fluorescein staining before and after 4 weeks applications of eye drops. Corneal sensitivity was measured by a modified Cochet-Bonnet esthesiometer for mice (Supplementary Fig. S1a). Results showed that SM-345431 significantly preserved murine corneal sensitivity (2.3 ± 0.3 mm in DED-SM-345431 group versus 1.4 ± 0.1 mm in DED-vehicle group, p = 0.004). There was no significant difference in non-DED mice (Fig. 1a). Decrease in tear volume was significantly suppressed in the DED-SM-345431 group (1.1 ± 0.1 mm versus 0.3 ± 0.1 mm in DED-vehicle group, p < 0.001). There was no significant difference in non-DED mice (Fig. 1b).

**Figure 2:**
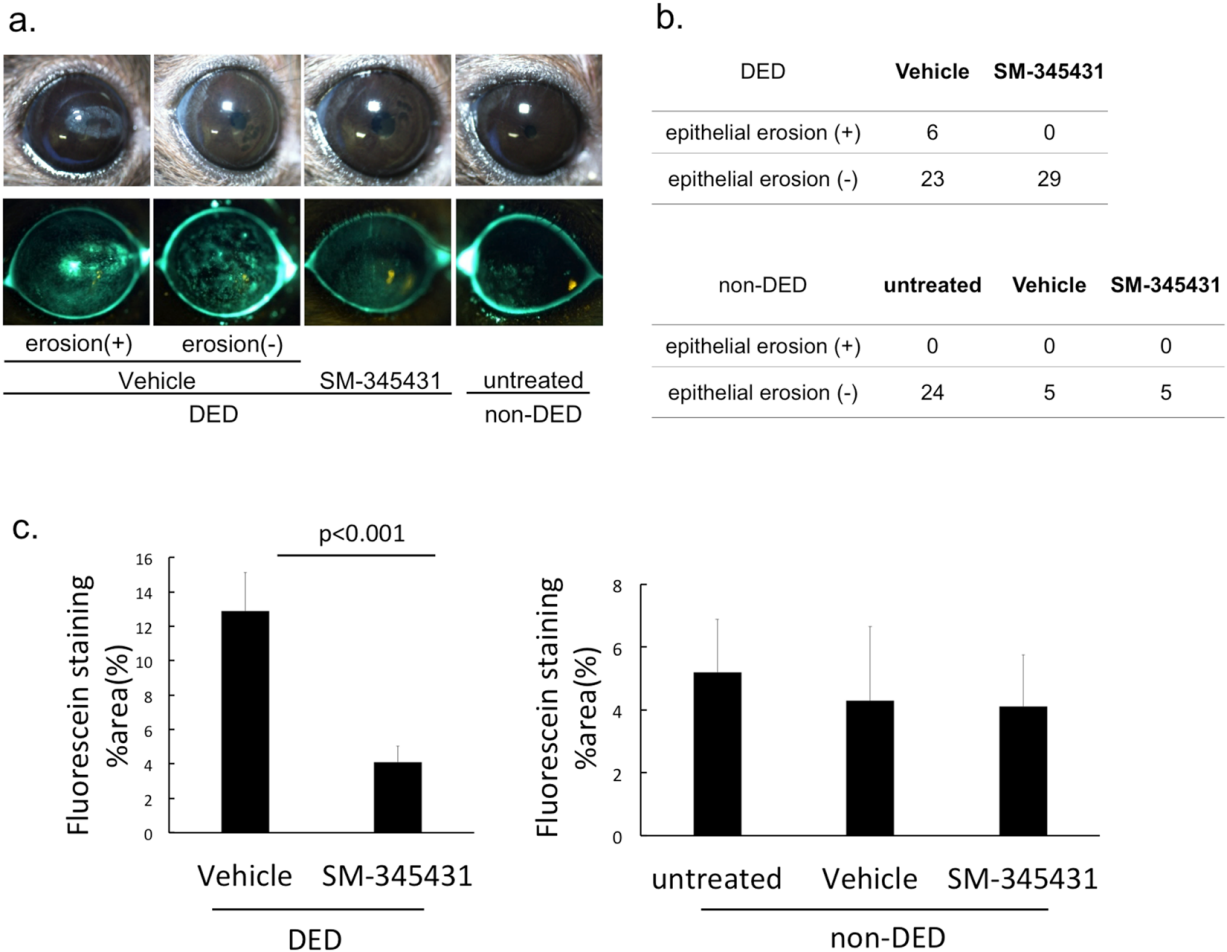
Corneal epithelial damage. (**a**) Bright field images (upper panel) and fluorescein staining (lower panel) after 4 weeks. (**b**) Numbers of corneas that developed epithelial erosions in DED groups (upper row) and non-DED groups (lower row). (**c**) Area of fluorescein staining relative to entire cornea (%). DED group (left), and non-DED group (right). DED groups were analysed by Student’s t-test (n = 15 mice/group) Non-DED groups were analysed by one way ANOVA and Bonferroni post hoc analysis, n = 5 mice/group. Graphs expressed as mean ± SD.

Furthermore, SM-345431 suppressed epithelial erosion formation (Fig. 2a). The incidence of epithelial erosion is shown in Fig. 2b. In the DED-vehicle group, corneal epithelial erosions developed in 6 out of 29 mice (21%), while none of the DED mice treated with SM-345431 developed an erosion (0%). Comparison among DED groups shows that the SM-345431 group significantly suppressed epithelial erosions following lacrimal gland extraction (p = 0.01, Fisher’s exact test, n = 29 mice/group).

DED-vehicle mice did not develop erosions, although some developed superficial punctate keratitis (Fig. 2a). We defined “erosion” as the complete loss of corneal epithelial cells, including epithelial basal cells. Superficial punctate keratitis is due to the cell loss of superficial squamous cells only.

Corneal fluorescein stained area was significantly reduced in the DED-SM-345431 group (4.1 ± 0.9% in DED-SM-345431 group versus 12.9 ± 2.2% in DED-vehicle group, p < 0.001). There was no significant difference in the non-DED groups (Fig. 2c).

### Denervation and morphological changes of the corneal sub-epithelial nerves

**Figure 3:**
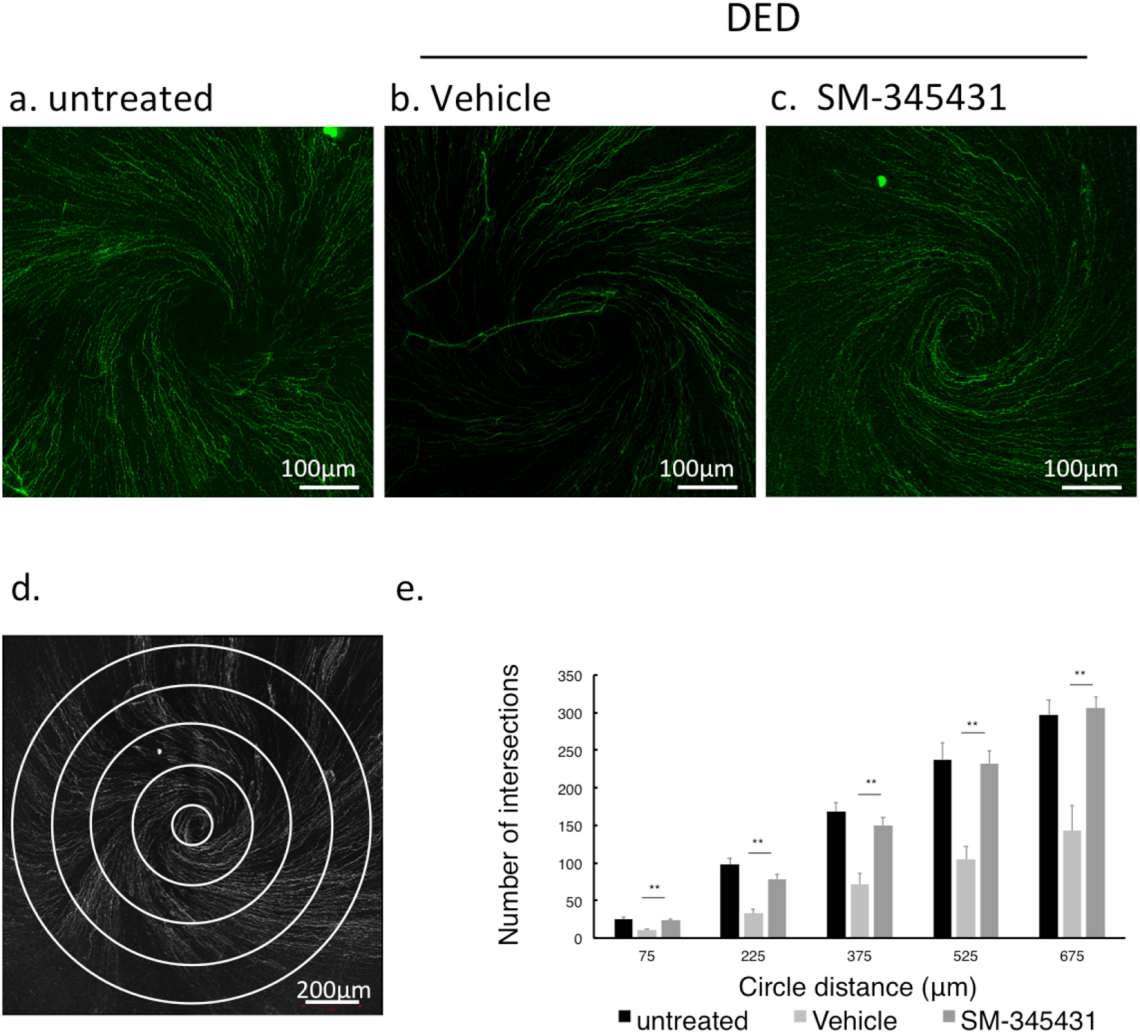
Corneal sub-epithelial nerve fibres. Each image was reconstructed from images taken by confocal microscopy using z-stack and maximum intensity projection. (**a**) non-DED untreated, (**b**) DED-vehicle, and (**c**) DED-SM-345431 whole-mounted corneas stained with rabbit monoclonal anti-TUJ-1 (βIII tubulin) antibody (green). (**d**) For Sholl analysis, five concentric circles (150 μm apart) were superimposed on each image. (**e**) The number of intersections were counted in each group. Statistical analysis was performed using ANOVA and Bonferroni post hoc analysis. Data expressed as mean ± SD; n = 5 (for non-DED untreated group), n = 10 (for DED-vehicle and DED-SM-345431 groups) mice/group, **P < 0.01.

Immunohistochemistry of βIII tubulin was done to identify the morphology of corneal nerve fibres on non-DED untreated group, and DED vehicle and SM-345431 group. Thick nerve trunks in the corneal stroma were observed extending toward the corneal epithelium. At the sub-epithelial level, nerve fibres become thinner and ran parallel to the basement membrane forming a vortex at the centre of the cornea. (Fig. 3a–c) In the DED-vehicle group, the sub-epithelial nerve plexus became less dense and lost characteristic fine brush-like fibres (Fig. 3b), while they were preserved in the DED-SM-345431 group (Fig. 3c). Sholl analysis was performed to quantitate nerve fibre change (Fig. 3d). Nerve density in the DED-SM-345431 group was significantly greater compared to the DED-vehicle group in all points (p < 0.001, one way ANOVA and Bonferroni post hoc). (Fig. 3e).

### Effect of SM-345431 on neovascularization

**Figure 4:**
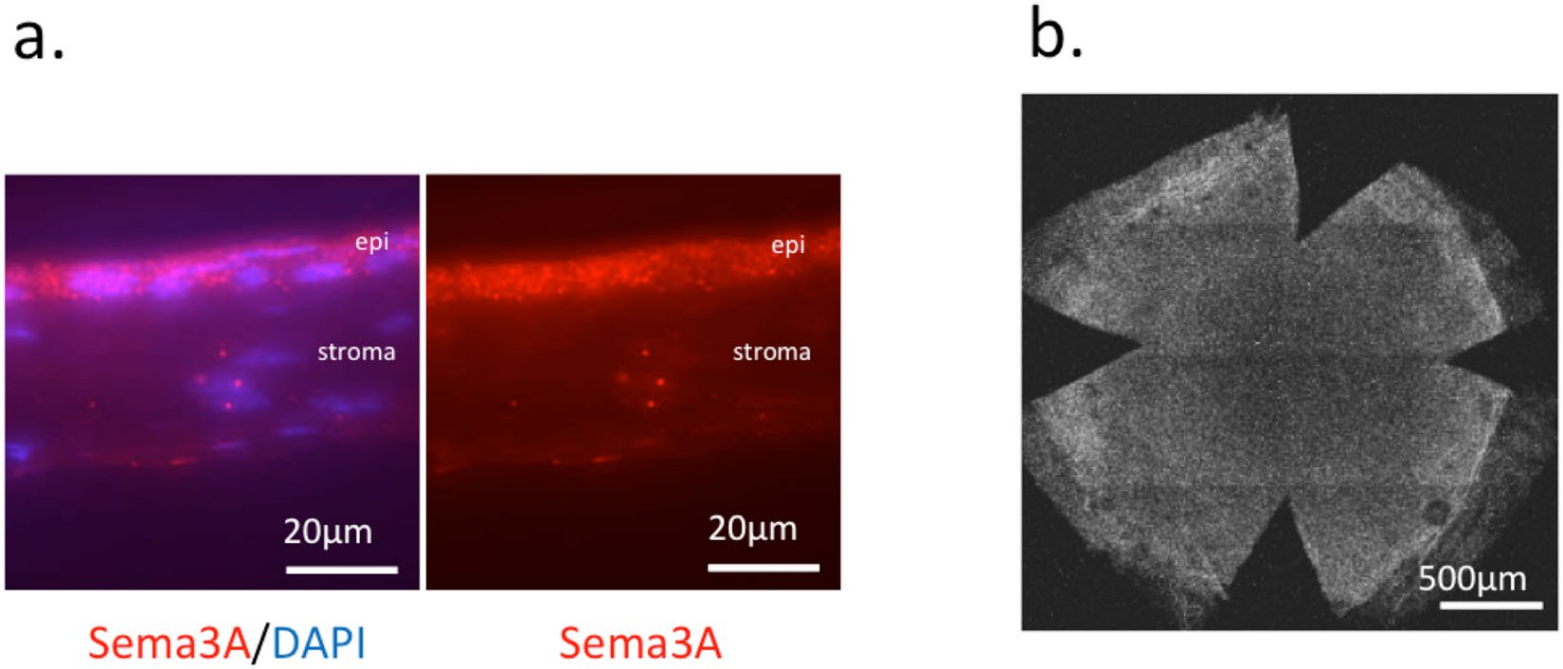
Sema3A expression and blood vessels in the corneal epithelium. (**a**) Sema3A (red) expression in corneal epithelium of non-DED untreated group. Nuclei were stained with DAPI. (**b**) Sema3A is also known to suppress VEGF-induced neovascularization. To examine possible angiogenesis by inhibiting Sema3A, CD31 was stained in whole mount cornea of SM-345431 group, using rat anti-CD31 antibody. Vessels were not observed crossing the corneal limbs.

The expression of Sema3A in corneal epithelium was confirmed by immunohistochemical staining of corneal cryosections of non-DED untreated group (Fig. 4a). Sema3A is also known as an inhibitor of VEGF-induced neovascularization by competing with VEGF for binding to their common receptor, neuropilin-1 (NP-1). Therefore, although neovascularization may be a side effect of Sema3A inhibition, we did not observe vessel growth into the cornea at concentrations used in our study. (Fig. 4b).

### Preserved TRPV1 expression in corneal epithelium by SM-345431 treatment

**Figure 5:**
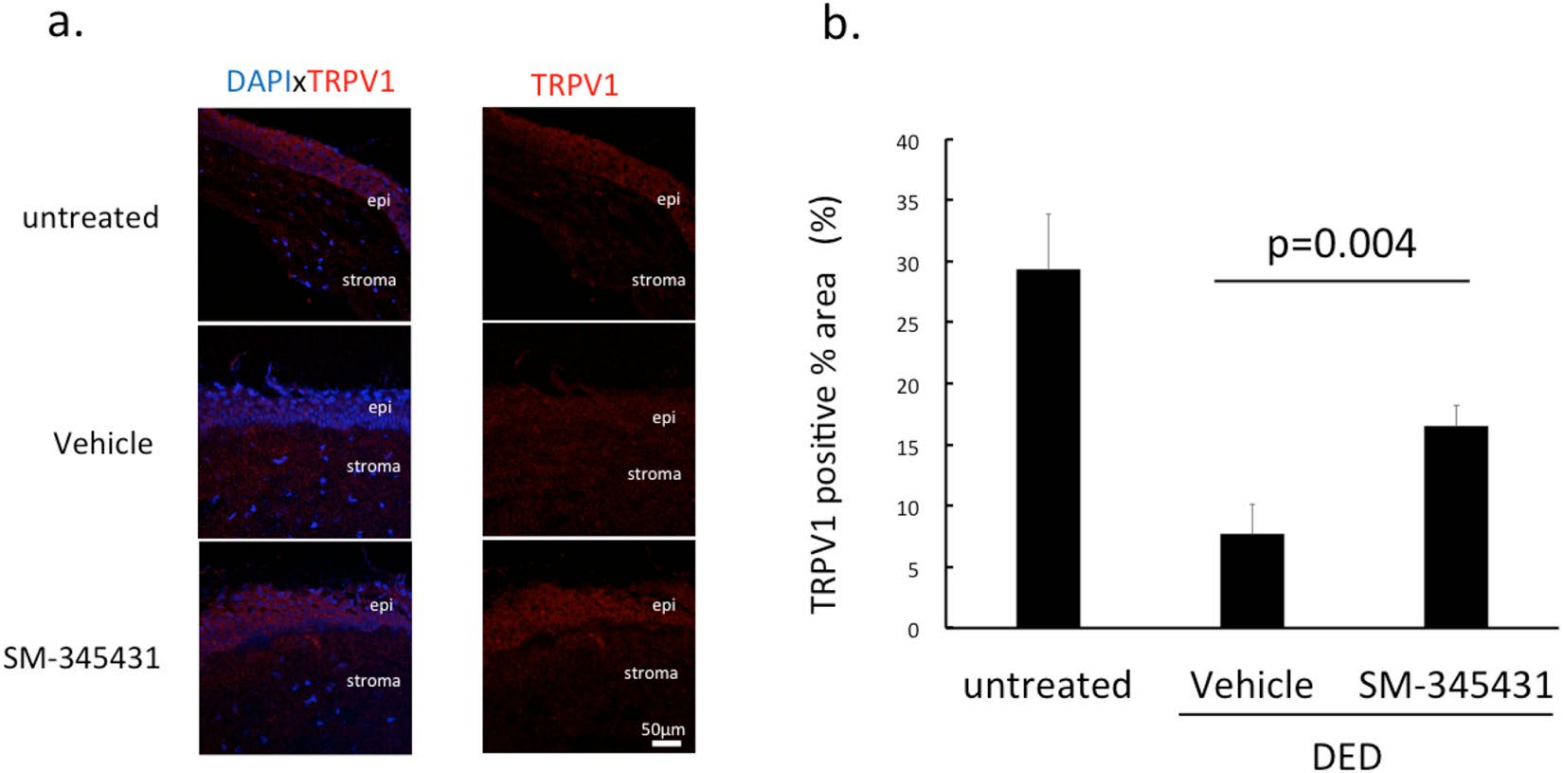
Quantitative analysis for TRPV1 expression in corneal epithelium. (**a**) Immunohistochemistry was performed on murine corneal sections. Tissues were stained with rabbit polyclonal anti- TRPV1 antibody (red) and DAPI (blue) captured at 40x magnification, under oil immersion. Images were analysed on Image J software by counting TRPV1 positive area in the corneal epithelium. (**b**) Graph shows TRPV1-positive area within the epithelium (%). Statistical analysis was performed using ANOVA and Bonferroni post hoc analysis. Data expressed as mean ± SD; n = 5 (for non-DED untreated group), n = 10 (for DED-vehicle and DED-SM-345431 groups) mice/group.

TRPV1 is a polymodal nociceptor expressed on sensory neurons. To evaluate the expression of TRPV1, we calculate the percentage of TRPV1 positive areas in the corneal epithelium using the ImageJ software (Fig. 5a). The expression of TRPV1 was significantly larger in the DED-SM-345431 group (16.5 ± 1.7%) compared to the DED-vehicle group (7.7 ± 2.4%) (p = 0.04, one way ANOVA and Bonferroni post hoc) (Fig. 5b).

## Discussion

Sema3A is an extracellular matrix molecule expressed in the cornea, where it is suggested to have a role in epithelial wound healing^20^. Sema3A suppresses axonal growth during development, as well as during wound healing in the adult. We previously reported that selective inhibition of Sema3A by SM-345431 enhanced nerve regeneration in a murine cornea transplantation model^19^. Several studies have reported that corneal nerve density is reduced in DED^1,2,7,21–28^. Our murine dry eye model of removing the lacrimal gland showed similar damage to the corneal nerves observed in humans. Compared to other murine dry eye models that involve the use of dry chambers^29^, our method reflects the pathology in human patients where tear secretion is decreased, and also preserves the contralateral eye as a normal control. Using this model, we showed that topical application of the Sema3A inhibitor, SM-345431, can prevent disruption of corneal nerves, maintain corneal sensation and tear volume. Furthermore, dry eye mice treated with SM-345431 did not form corneal epithelial erosions as in untreated mice.

The cornea is one of the most densely innervated tissues in the body. The sub-epithelial nerve plexus of the cornea originates from the peripheral stromal nerves^30^. Branches of stromal nerves in sub-epithelial nerve plexus penetrate Bowman’s membrane and contribute to the sub-epithelial nerve plexus with a well-defined vortex pattern. Corneal nerves have two types of nociceptive fibres, A-delta and C fibre^31^. Almost all stromal nerves are A-delta fibres with myelinated straight axons, and the sub-epithelial nerves are C fibres with no myelination. After branching from thick A-delta fibres at the level of sub-epithelial nerve plexus, bared unmyelinated C fibres go upward and terminate just beneath the corneal epithelial surface as free nerve endings^32^. These nociceptive fibres enable cornea to respond to mechanical, thermal, and chemical stimuli as described by Belmonte *et al*.^33–35^. These sub-epithelial nerves are rapidly degraded when the cornea suffers injury^36^.

Interestingly, we found that mice that did not suffer epithelial erosions did not show vast nerve damage compared to mice with erosions. Therefore, epithelial damage may precede nerve damage in our model. However, since corneal sensation was preserved with the use of SM-345431, even in mice with no apparent damage in sub-epithelial nerves, we examined the expression of nociceptors expressed in the nerve endings. TRP (transient receptor potential) family members are ligand-gated ion channels, which act as the molecular detectors of stimuli^37^. TRPV1 is a polymodal nociceptor expressed on sensory neurons of the corneal epithelium. We observed a decrease in TRPV1 expression in DED model mice, which was preserved with SM-345431 application. This suggests that DED following lacrimal gland extraction leads to the loss of TRPV1, explaining the loss of corneal sensation in mice without erosions. Further insult to the cornea leads to erosions and then visible damage to the sub-epithelial nerve plexus.

While SM-345431 had a definitive protective effect on the integrity of the corneal epithelium and sub-epithelial nerves in our DED model, the precise mechanisms involved still need to be clarified. In our previous publication using a mouse corneal transplantation model, SM-345431 clearly enhanced the regeneration of host nerves within the donor cornea^19^. However, our current DED model showed that SM-345431 had protective effects without evident nerve damage. One possibility is that SM-345431 may have anti-inflammatory properties that suppress the damage to epithelium and nerves due to inflammation following dry eye. Sema3A also acts to recruit inflammatory cells^38^, and SM-345431 may block this aspect of Sema3A in addition to neural effects. Further studies are underway to test this hypothesis.

The response of nociceptors and nerves to inflammation is complex. Inflammation may act on TRPV1 to produce pathologically sensitized signalling^39,40^. A previous study showed that blocking of TRPV1 suppressed inflammation and fibrosis/scarring during healing of murine cornea^41^. However, mice treated with SM-345431 did not show signs of eye rubbing associated with irritation or pain. Furthermore, SM-345431 inhibited damage to the epithelial barrier demonstrated by the uptake of fluorescein dye. SM-345431 is also reported to have weak matrix metalloproteinases (MMP) inhibition activity^18^. Inhibition of MMP activity may also be a possible mechanism since MMP increases in tears of patients with ocular surface disease^42^, as well as in experimental dry eye mouse models^43^.

Damage to the epithelial barrier further leads to epithelial erosions causing extensive damage to the sub-epithelial nerve plexus. This may initiate a vicious cycle that further exacerbates DED. A previous study showed that DED patients with near-normal corneal nerves and functionally intact nerve endings were found to responded to therapy, while patients with severely damaged corneal nerves did not show post-treatment improvement in a prospective randomized clinical trial of a combination of artificial tears and low dose steroids^1^. Early intervention to preserve epithelial and nerve integrity may be one of the keys to prevent severe dry eye.

In summary, our study demonstrated that topical application of a Sema3A inhibitor preserved corneal sensitivity and tear volume, reduced corneal epithelial damage and preserved corneal nerve density and intraepithelial expression of TRPV1 in a DED mouse model. Although further studies are necessary, our results suggest that topical Sema3A may be an effective treatment of clinical DED.

## Methods

### Animal models

C57BL/6JJc1 mice, all 9-10-week-old females, were purchased from CLEA Japan Inc. (Tokyo, Japan). Experimental protocol was approved by the Experimental Animal Committee of Keio University, School of Medicine (Approval Number. 08050-(8)). All animal experiments were performed in accordance with the guidelines of experimental animal care committee of Keio University, School of Medicine. Dry eye model (DED) mice were prepared by excising the extra-orbital lacrimal gland of right eye. Each mouse was anesthetized by intra muscular injection of a mixture of 0.2 mg midazolam, 0.1875 mg medetomidine, and 0.25 mg butorphanol prior to surgical procedures. DED mice were divided to two groups, and PBS (vehicle) or SM-345431 eye-drops were applied 3 times a day for 4 weeks immediately after lacrimal grand extraction. On the other hand, non-DED (intact lacrimal gland) mice were used to test the potential iatrogenic effect of the SM-345431. Non-DED mice were divided to three groups, untreated (no eye drops), vehicle, and SM-345431. Vehicle or SM-345431 eye-drops were applied 3 times a day for 4 weeks.

### Sema3A inhibitor

SM-345431 (vinaxanthone), a Sema3A inhibitor isolated from a fungus *penicillium* sp. Strain SPF-3059 was provided from Sumitomo Dainippon Pharma Co., Ltd, Osaka, Japan. Detailed isolation of SM-216289, a similar compound to SM-345431 used in this study was described previously^17,44^. Briefly, the fermented broth of *penicillium* sp. Strain SPF-3059 cultured for 8 days with reciprocal shaking was centrifuged at 10,000*g* for 10 minutes. The precipitated cells were extracted with acetone and the supernatant was extracted with 1% formic acid-ethyl acetate. Both acidic ethyl acetate extracts were combined and purified using a combination of gel filtration chromatography and reverse-phase high pressure liquid chromatography to isolate SM-345431. According to the previous pharmacological properties of SM-216289, we performed preliminary experiments using 0.5 mg/ml and 2.0 mg/ml of SM-345431, and found that the results were similar. We therefore used 0.5 mg/ml for all experiments.

### Corneal sensitivity measurement

To measure murine corneal sensitivity, we performed a modified blink test. Since murine corneas are smaller than in humans, using a standard Cochet-Bonnet esthesiometer (0.12 mm diameter) was not accurate. Therefore, we used a modified Cochet-Bonnet esthesiometer (Luneau Ophtalmologie, Chartres Cedex, France) using a thinner nylon filament of 0.03 mm diameter to measure the threshold to induce blinking in mice (Supplementary Fig. S1a). Data are expressed as the length of filament (cm) required to cause blinking.

### Tear volume measurement

The phenol red thread test, ZONE-QUICK® (AYUMI Pharmaceutical Corporation, Tokyo, Japan) was used for measurement of murine tear volume^45^. (Supplementary Fig. S1b). The tear volume, expressed in millimetres of thread turned red, was recorded.

### Corneal fluorescein staining

Murine corneal fluorescein staining was performed by applying 1 μl of fluorescein solution on the cornea using a micropipette, followed by three times wash with 1 ml of PBS under anaesthesia. Fluorescence imaging of stained murine corneas were performed using a fluorescent stereo-microscope (Leica M165 FC) with a CCD camera (Leica DFC310 FX). All images were acquired using the same intensity settings. Images were analysed using ImageJ software (https://imagej.nih.gov/ij/; provided in the public domain by the National Institution of Health, Bethesda, MD, USA) and the percentage of fluorescein-stained area was calculated.

### Immunohistochemistry

After 4 weeks of treatment, mice were sacrificed by cervical dislocation. For corneal whole-mount immunohistochemistry, eye balls were fixed with 1% formaldehyde, 2 mM MgCl_2_, 5 mM EGTA, and 0.02% NP-40, in 1x PBS for 1 hour and 15 minutes at 4 °C, immediately after excision. Details of the protocol have been reported previously by Ganji *et al*.^36^. For immunohistochemistry with cryosections, 10 μm sections were prepared from fresh frozen corneas and fixed with 4% paraformaldehyde (PFA; Wako Ltd, Osaka, Japan) for 5 minutes. After three times washes with PBS, sections were incubated in fixative (Morphosave; Ventana Medical Systems, Tucson, AZ) for 15 minutes. Blocking was performed with 10% normal donkey serum with PBS containing 0.3% Triton-X for 30 minutes. Sections were incubated with primary antibodies for 1 hour at room temperature. After three time washes with PBS, sections were incubated with secondary antibodies for 1 hour at room temperature. After three time washes with PBS, the sections were mounted with Permafluor (Beckman Coulter Inc., Miami, FL) containing DAPI diluted 1:1000.

Details of the primary and second antibodies used in this study are shown in Table 1.

**Table 1:** Primary and secondary antibodies used in this study.

Primary antibody	Manufacturer	Dilution used
βIII tubulin	Cell Signaling TECHNOLOGY, Massachusetts, U.S.; rabbit monoclonal; #5568	1:400
Semaphorin 3A	Abcam, Cambridge, UK; rabbit polyclonal; #23393	1:50
CD31	RD biosciences, San Diego, U.S.; rat; #557355	1:50
TRPV1	Abcam, Cambridge, UK; rabbit polyclonal; #74855	1:300
Secondary antibody	Manufacturer	Dilution used
Donkey anti-rabbit Alexa 555	Abcam, Cambridge, UK; #150074	1:500
Donkey anti-rabbit FITC	Jackson immuno research, PA, U.S.; #711-095-152	1:200
Donkey anti-rat FITC	Jackson immuno research, PA, U.S.; #711-095-153	1:200

### Immunofluorescence Microscopy

Immunostained nerve fibres in the corneal epithelium were detected by confocal microscopy (LSM710, Carl Zeiss Microscopy GmbH, Germany). An argon laser, a HeNe laser, and a blue diode laser were used for FITC (488 nm laser line excitation), Alexa Flour 555 (543 nm laser line excitation), and DAPI (405 nm laser line excitation), respectively. All images were acquired using the same intensity setting. LSM Software ZEN 2011(Carl Zeiss) was used to acquire images, fuse the adjacent tiles with Tile Scan, and produce maximum intensity projection from z-stack images.

### Image data analysis

We applied sholl analysis to quantitate differences in corneal nerve fibre density^36,46^. The intersections of nerve fibres and five concentric circles (150 μm apart) were counted manually (Fig. 3d,e).

Quantitative data are presented as mean ± SD. Values were analysed using Student’s t-test two-tailed for 2 groups, one way ANOVA test for 3 groups, and Fisher’s exact test with two-tailed for DED groups in Fig. 2b. P values < 0.05 were considered statically significant for all comparisons.

## Electronic supplementary material

Supporting information

## References

[1] Kheirkhah A (2015). Effects of corneal nerve density on the response to treatment in dry eye disease. Ophthalmology.

[2] Shetty R (2016). Corneal Dendritic Cell Density Is Associated with Subbasal Nerve Plexus Features, Ocular Surface Disease Index, and Serum Vitamin D in Evaporative Dry Eye Disease. Biomed Res Int.

[3] Schein OD, Munoz B, Tielsch JM, Bandeen-Roche K, West S (1997). Prevalence of dry eye among the elderly. Am J Ophthalmol.

[4] Moss SE, Klein R, Klein BE (2008). Long-term incidence of dry eye in an older population. Optom Vis Sci.

[5] Jie Y, Xu L, Wu YY, Jonas JB (2009). Prevalence of dry eye among adult Chinese in the Beijing Eye Study. Eye (Lond).

[6] Han SB (2011). Prevalence of dry eye disease in an elderly Korean population. Arch Ophthalmol.

[7] Labbe A (2013). Corneal nerve structure and function in patients with non-sjogren dry eye: clinical correlations. Investigative ophthalmology & visual science.

[8] Dastjerdi MH, Dana R (2009). Corneal nerve alterations in dry eye-associated ocular surface disease. Int Ophthalmol Clin.

[9] Ambrosio R, Tervo T, Wilson SE (2008). LASIK-associated dry eye and neurotrophic epitheliopathy: pathophysiology and strategies for prevention and treatment. J Refract Surg.

[10] Dickendesher, T. L., Duan, Y. & Giger, R. J. In *Cellular Migration and Formation of Neuronal Connections* (ed. Pasko, Rakic) 151–175 (Academic Press, 2013).

[11] Luo Y, Raible D, Raper JA (1993). Collapsin: a protein in brain that induces the collapse and paralysis of neuronal growth cones. Cell.

[12] Wild JR, Staton CA, Chapple K, Corfe BM (2012). Neuropilins: expression and roles in the epithelium. Int J Exp Pathol.

[13] De Winter F (2002). Injury-induced class 3 semaphorin expression in the rat spinal cord. Exp Neurol.

[14] Pasterkamp RJ (1999). Expression of the Gene Encoding the Chemorepellent Semaphorin III Is Induced in the Fibroblast Component of Neural Scar Tissue Formed Following Injuries of Adult But Not Neonatal CNS. Molecular and Cellular Neuroscience.

[15] Taniguchi M (1997). Disruption of semaphorin III/D gene causes severe abnormality in peripheral nerve projection. Neuron.

[16] Tanelian DL, Barry MA, Johnston SA, Le T, Smith GM (1997). Semaphorin III can repulse and inhibit adult sensory afferents *in vivo*. Nat Med.

[17] Kaneko S (2006). A selective Sema3A inhibitor enhances regenerative responses and functional recovery of the injured spinal cord. Nat Med.

[18] Zhang L (2014). Rewiring of regenerated axons by combining treadmill training with semaphorin3A inhibition. Mol Brain.

[19] Omoto M (2012). The semaphorin 3A inhibitor SM-345431 accelerates peripheral nerve regeneration and sensitivity in a murine corneal transplantation model. PLoS One.

[20] Morishige N, Ko JA, Morita Y, Nishida T (2010). Expression of semaphorin 3A in the rat corneal epithelium during wound healing. Biochemical and biophysical research communications.

[21] Villani E (2008). Corneal involvement in rheumatoid arthritis: An *in vivo* confocal study. Investigative Ophthalmology and Visual Science.

[22] Villani E, Galimberti D, Viola F, Mapelli C, Ratiglia R (2007). The cornea in Sjögren’s syndrome: An *in vivo* confocal study. Investigative Ophthalmology and Visual Science.

[23] Villani E (2013). *In vivo* confocal evaluation of the ocular surface morpho-functional unit in dry eye. Optometry and Vision Science.

[24] Hamrah P (2017). Corneal Nerve and Epithelial Cell Alterations in Corneal Allodynia: An *In Vivo* Confocal Microscopy Case Series. Ocul Surf.

[25] Erdélyi B, Kraak R, Zhivov A, Guthoff R, Németh J (2007). *In vivo* confocal laser scanning microscopy of the cornea in dry eye. Graefe’s Archive for Clinical and Experimental Ophthalmology.

[26] del Castillo JMBT, Wasfy MAS, Fernandez C, Garcia-Sanchez J (2004). An *In Vivo* Confocal Masked Study on Corneal Epithelium and Subbasal Nerves in Patients with Dry Eye. Investigative ophthalmology & visual science.

[27] Benítez-Del-Castillo JM (2007). Relation between corneal innervation with confocal microscopy and corneal sensitivity with noncontact esthesiometry in patients with dry eye. Investigative Ophthalmology and Visual Science.

[28] Zhang X (2011). Tear dynamics and corneal confocal microscopy of subjects with mild self-reported office dry eye. Ophthalmology.

[29] Barabino S (2005). The controlled-environment chamber: a new mouse model of dry eye. Investigative ophthalmology & visual science.

[30] Ivanusic JJ, Wood RJ, Brock JA (2013). Sensory and sympathetic innervation of the mouse and guinea pig corneal epithelium. J Comp Neurol.

[31] Muller LJ, Vrensen GF, Pels L, Cardozo BN, Willekens B (1997). Architecture of human corneal nerves. Investigative ophthalmology & visual science.

[32] Shaheen BS, Bakir M, Jain S (2014). Corneal nerves in health and disease. Survey of Ophthalmology.

[33] Belmonte C, Garcia-Hirschfeld J, Gallar J (1997). Neurobiology of ocular pain. Progress in Retinal and Eye Research.

[34] Belmonte C, Acosta MC, Gallar J (2004). Neural basis of sensation in intact and injured corneas. Exp Eye Res.

[35] Belmonte C, Acosta MC, Merayo-Lloves J, Gallar J (2015). What Causes EyePain?. Curr Ophthalmol Rep.

[36] Pajoohesh-Ganji A (2015). Partial denervation of sub-basal axons persists following debridement wounds to the mouse cornea. Lab Invest.

[37] Dai Y (2016). TRPs and pain. Semin Immunopathol.

[38] Ji JD, Park-Min KH, Ivashkiv LB (2009). Expression and function of semaphorin 3A and its receptors in human monocyte-derived macrophages. Human immunology.

[39] Murata Y, Masuko S (2006). Peripheral and central distribution of TRPV1, substance P and CGRP of rat corneal neurons. Brain Res.

[40] Petho G, Reeh PW (2012). Sensory and signaling mechanisms of bradykinin, eicosanoids, platelet-activating factor, and nitric oxide in peripheral nociceptors. Physiol Rev.

[41] Okada Y (2011). TRPV1 involvement in inflammatory tissue fibrosis in mice. Am J Pathol.

[42] Chotikavanich S (2009). Production and activity of matrix metalloproteinase-9 on the ocular surface increase in dysfunctional tear syndrome. Investigative ophthalmology & visual science.

[43] Corrales RM (2006). Desiccating stress stimulates expression of matrix metalloproteinases by the corneal epithelium. Investigative ophthalmology & visual science.

[44] Kumagai K, Hosotani N, Kikuchi K, Kimura T, Saji I (2003). Xanthofulvin, a novel semaphorin inhibitor produced by a strain of Penicillium. J Antibiot (Tokyo).

[45] Yoon KC (2011). Tear production and ocular surface changes in experimental dry eye after elimination of desiccating stress. Investigative ophthalmology & visual science.

[46] Libersat F (2005). Maturation of dendritic architecture: lessons from insect identified neurons. Journal of neurobiology.

