# Supplementary material for: The Semaphorin 3A inhibitor SM-345431 preserves corneal nerve and epithelial integrity in a murine dry eye model
Source: Sci Rep. 2017 Nov 14;7:15584. doi: 10.1038/s41598-017-15682-1 (PMC5686158; doi:10.1038/s41598-017-15682-1)
Supplement: Supplementary file 1 — Supporting information [file 41598_2017_15682_MOESM1_ESM.pdf]

Title;

The Semaphorin 3A inhibitor SM-345431 preserves corneal nerve and epithelial integrity in a murine dry eye model.

Running title;

Semaphorin 3A inhibition preserves corneal nerves

Names of authors;

Risa Yamazaki[1], Katsuya Yamazoe[1], Satoru Yoshida[1 3], Shin Hatou[1], Emi Inagaki[1], Hideyuki Okano[1 2], Kazuo Tsubota[1], and Shigeto Shimmura[1]*.

Names of institution;

[1] Department of Ophthalmology, Keio university school of medicine, Tokyo, Japan

[2] Department of Physiology, Keio university school of medicine, Tokyo, Japan

[3] Centre for Rare Disease Research, National Institutes of Biomedical Innovation, Health and Nutrition, Osaka, Japan

*Corresponding Information;

shige@z8.keio.jp

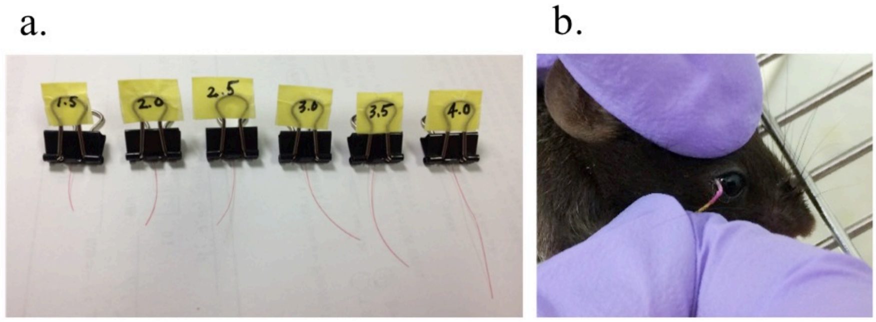

Supplementary Figure 1. Dry eye model and modified Cochet-Bonnet esthesiometer for mice

(a) We used thin nylon filaments (0.03mm diameter, quarter of normal Cochet-Bonnet esthesiometer) to measure the threshold to induce blinking in mice. (b) Applying ZONE-QUICK® for measurement of tear volume.

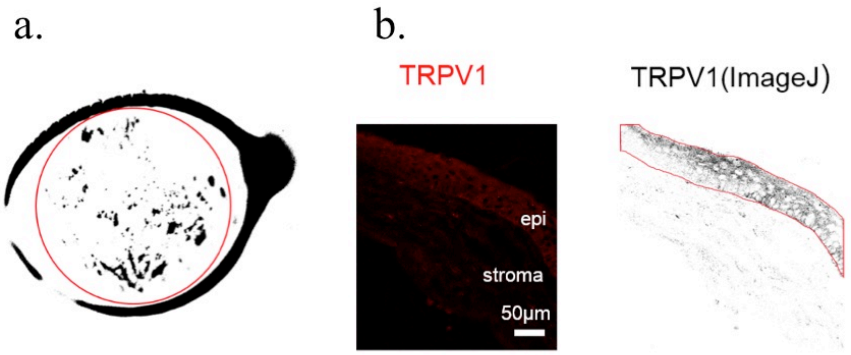

Supplementary Figure 2. Image analyses using Image J software

(a) The percentage of fluorescein-stained area was calculated using the Image J software under the same threshold in all images. The area within the red circle with a diameter adjusted to the vertical corneal diameter was used for analysis. (b) To analyse the expression of TRPV1 in corneal epithelium, the corneal epithelial area was selected (red) and the percentage of TRPV1-stained area was calculated under the same threshold settings.
